# From the origin of life to pandemics: emergent phenomena in complex systems

**DOI:** 10.1098/rsta.2020.0410

**Published:** 2022-07-11

**Authors:** Oriol Artime, Manlio De Domenico

**Affiliations:** ^1^ Fondazione Bruno Kessler, Via Sommarive 18, Povo, TN 38123, Italy; ^2^ Department of Physics and Astronomy ‘Galileo Galilei’, University of Padua, Padova, Veneto, Italy

**Keywords:** emergence, complexity, complex systems

## Abstract

When a large number of similar entities interact among each other and with their environment at a low scale, unexpected outcomes at higher spatio-temporal scales might spontaneously arise. This non-trivial phenomenon, known as emergence, characterizes a broad range of distinct complex systems—from physical to biological and social—and is often related to collective behaviour. It is ubiquitous, from non-living entities such as oscillators that under specific conditions synchronize, to living ones, such as birds flocking or fish schooling. Despite the ample phenomenological evidence of the existence of systems’ emergent properties, central theoretical questions to the study of emergence remain unanswered, such as the lack of a widely accepted, rigorous definition of the phenomenon or the identification of the essential physical conditions that favour emergence. We offer here a general overview of the phenomenon of emergence and sketch current and future challenges on the topic. Our short review also serves as an introduction to the theme issue *Emergent phenomena in complex physical and socio-technical systems: from cells to societies*, where we provide a synthesis of the contents tackled in the issue and outline how they relate to these challenges, spanning from current advances in our understanding on the origin of life to the large-scale propagation of infectious diseases.

This article is part of the theme issue ‘Emergent phenomena in complex physical and socio-technical systems: from cells to societies’.

## 1. Introduction

*d oijw ao o fyrg bafjdsdpw dweoda wdhao jrfgb sag wdgy d ias dsih sig qqpdjwe fjrfb dvvs*. In the previous sentence, each character is randomly generated: the way characters cluster together, while being separated by spaces, are usually interpreted as words and sequences of words used to transmit information, e.g. a message. However, there is apparently no intelligible knowledge that can be extracted from the above example: from the perspective of the reader there is a lack of those familiar patterns that one routinely uses to communicate and expects to find with respect to some prior (e.g. a scientific paper written in English). Concisely, we can argue that there is no concept or knowledge in a single character: a sequence of characters (i.e. a word) and a sequence of words (i.e. a sentence) exchanged between a sender and a receiver becomes meaningful when both of them spontaneously start to use repeating patterns which they identify as meaningful. In other words, language is an emergent phenomenon requiring a symbolic representation for units (i.e. the characters) and their interactions (i.e. special sequences of characters).

Rather intriguingly, such a *spontaneous appearance* of meaningful structures in space and time is a ubiquitous phenomenon observed from the microscopic scale—e.g. in molecular interactions within a cell—to the macroscopic one—e.g. the cosmic web, in complex adaptive matter [1]. In the following, we will briefly review the phenomenology concerning emergent phenomena and provide an operational definition of emergence as a hallmark of complexity that can be applied to a variety of complex systems, regardless of whether they are natural, social or artificial.

## 2. A brief historical overview

A primordial concept can be already found in the ‘Metaphysics’ written by the ancient Greek philosopher Aristotle, where it is argued that a totality is something besides the parts. The same concept, but with a slightly different meaning, can be found in Gestalt psychology, based on the intuition that organisms do not merely perceive individual components but entire patterns or configurations: in a nutshell, ‘the whole is other than the sum of the parts’ [2], and a similar concept appears in the work by William M. Wheeler in 1926 [3,4]. The concept has been invoked by emergentist philosophers such as Samuel Alexander and Charlie Dunbar Broad in contrast to reductionism in the 1920s, although a more formal approach was developed by the pioneers of cybernetics in the 1940s. In fact, cybernetics deals with systems and their causal feedback loops: among its founders there were Norbert Wiener and John von Neumann, the latter being the first to propose cellular automata and a universal constructor, both concepts strictly related to emergence. Ludwig von Bertalanffy, in particular, was among the founders of general systems theory, providing the first mathematical ground to describe the complexity observed in biological and social systems [5,6].

It was during the 1970s that the Nobel Laureate Phil Anderson warned against the perils of reductionism. In [7], he gave specific examples where reductionist thinking fails and highlighted the fact that the most fundamental physical laws were unable to explain new properties and behaviours arising in the assembly of a large number of units obeying those fundamental laws. An important consequence of this is that he opened the door to the existence of fundamental laws at different levels of complexity, e.g. the objects of study in biology do not follow the laws of chemistry, likewise the objects of study in chemistry do not follow the laws of particle physics. Anderson argued that, even if we are looking at a single level of complexity in this hierarchy, it is via a process of *symmetry breaking* that the state of a large system composed of many entities might not follow the rules of the fundamental laws that the entities themselves follow. Hence, the appearance of new properties is intimately linked with the disappearance of the symmetries of a system, be they spatial, temporal, informational, etc. As a particular example of emergence by symmetry breaking, we can mention the formation of complex spatio-temporal patterns in dissipative systems, where the isotropic symmetry that one would expect from thermodynamics is broken [8]. It is noteworthy that the development of this theory was contemporary to Anderson’s seminal paper, and earned Ilya Prigogine the Nobel prize in 1977. After Anderson’s illuminating article, we have witnessed an upsurge of contributions that kept exploring the implications of this concept, both at the theoretical and applied levels, see e.g. [9] for a compilation of some of these.

One of the directions that Anderson pointed out as interesting to explore is the emergence in living beings. The origin of life can be seen as an instance of enormous complexity whose inception is based on the interaction of cells that perform simple, decentralized tasks. One way to approach this is via computer simulations. Indeed, during the 1980s, computers became widespread and thus propelled the exploration of emergent phenomena from a computational point of view. It is inevitable that we mention here the influential investigations of Stephen Wolfram in cellular automata. He introduced the numbering scheme still used nowadays [10] and, among others breakthroughs, he conjectured in 1985 that *Rule 110 cellular automaton* was Turing-complete—formally proven almost two decades later [11]. In recent years, the research in emergence of life has kept advancing and has incorporated more and more biological and molecular mechanisms.

The last few decades have been characterized by the realization that many central problems in physics, but also in other branches of sciences, could be understood as emergent phenomena, such as superfluidity or the fractional quantum Hall effect [12] which led Robert Laughlin to be awarded the Nobel Prize for Physics in 1998. The study of emergent phenomena has been made even more popular by Murray Gell-Mann (Nobel Prize for Physics in 1969) [13] and has become more and more an interdisciplinary endeavour having found in the wide umbrella of complexity science a substrate to develop [14]. Nowadays, efforts go in the directions of identifying, characterizing and understanding such phenomena, with a balanced combination of analytical, computational and experimental techniques, as well as providing a formal theory of emergence, with considerable developments made thanks to information-theoretic tools. The last great news for the field, which is far from being a fringe theory, concerns the Nobel Prize for Physics awarded to Giorgio Parisi in 2021, for his studies of disordered physical complex systems and their fluctuations.

## 3. What is emergence and why does it matter?

Simple systems are mostly characterized by the fact that the properties of the whole can be understood, deduced or predicted from the analysis of their components in isolation, their addition or their aggregation: in practice, macroscopic observables can be deduced from microscopic ones. From this observation, it is clear that in order to characterize an emergent phenomenon one needs at least two well separated scales—for instance defined in terms of energy or in space and time—and one external observer able to identify meaningful patterns, and measure them in terms of information, appearing at one scale but not at the others. Let us consider, for instance, the mass M of composite objects like a chair, which consists of distinct parts with a mass $m_{i}$ (i=1,2,…,n): the overall mass can be simply obtained by summing up the mass of each component as $M=\sum_{i}m_{i}$. At a smaller scale, let us say at a molecular one, a similar approach leads to a similar result. At the lowest scale, like the one of atomic nuclei, one could argue that the same approach would still lead to a similar result, although this is not exactly the case because the strong interaction which combines protons and neutrons—i.e. the nuclear force—is responsible for a mass defect which is converted into binding energy according to the mass-energy equivalence. The mass is an interesting property, since at spatial scales much larger than the atomic one the linear approximation applies very well, while at the lowest scale it does not. At a fundamental level, like in quantum field theory, mass allows us to measure the coupling of a particle with the Higgs field but it is not considered an emergent property, although the issue has been debated [15]. It is also interesting that at the nuclear scale, the presence of interactions between fundamental constituents is able to generate a deviation from the naive expectation that a simple summation applies. It is worth remarking that the mass property can be defined at the level of a single particle as well as at the level of an aggregation of particles, regardless if they are interacting or not. This is also the case for other physical properties such as the spin, for instance.

However, there are properties that cannot be defined at the level of a single unit, whether a particle, a cell or an individual: such properties are meaningful only at some scale larger than the one defining a single unit. When this is the case, the corresponding phenomena are usually referred to as *emergent*: emergence is considered a fundamental feature of complex adaptive matter, transcending the traditional frontiers of theoretical physics and becoming a landmark in a broad spectrum of disciplines, from biology to neuroscience, from system ecology to economics. In the following, we will briefly review a broad class of complex systems across a variety of disciplines, starting from the quantum realm and then moving to non-quantum systems, including physics, biology, ecology, social and urban sciences. A special focus will be given to results obtained from network science, where several emergent properties are related to non-trivial structure, non-trivial dynamics or their interplay.

### (a) Emergence in quantum physical systems

Quantum mechanics is responsible for many fascinating emergent phenomena, such as localization and superconductivity. Regarding the former, it was during the 1950s that Phil Anderson suggested that in a sufficiently large lattice, a sufficient amount of disorder prevents standard diffusion of waves [16], which is a setup that can be effectively realized by means of impurities or defects in semiconductors. As per superconductivity, we know that a superconductor is a material where the collective behaviour of particles spontaneously emerge at a characteristic critical temperature: below such a temperature, the material does not exhibit electrical resistance, making these materials suitable for dissipation-free applications. Although some properties are material dependent, all superconductors break the U(1)-gauge symmetry down to $Z_{2}$ leading to universal properties such as the Meissner–Ochsenfeld effect and off-diagonal long-range order. In condensed matter physics, the origin of this phenomenon can be explained by the theory proposed by Bardeen, Cooper and Schrieffer, arguing that pairs of fermions, such as electrons, condensate into strongly interacting particles in the same ground quantum state—known as Cooper pairs—at low temperatures [17]. We refer the interested reader to a recent collection about emergent superconductivity [18].

At a larger scale, let us consider the case of two or more superconductors placed close enough to each other to be weakly coupled. The behaviour of the overall system was unexpected in the 1960s: known as the Josephson effect, the production of a supercurrent in the absence of voltage was first predicted by Brian Josephson in 1962 and later observed experimentally [19]. Such a phenomenon cannot be deduced from the knowledge of each superconductor in isolation: only the presence of weak coupling allows for the spontaneous appearance of such a collective behaviour. The quantum Hall effect, i.e. the quantized version of the Hall effect observed in systems at low temperatures, is another emergent phenomenon due to collective behaviour [12,20,21], together with the fractional quantum Hall effect [22–24].

### (b) Emergence in classical physical, non-living, systems

Since the pioneering work of Phil Anderson—using the mechanism of symmetry breaking to argue against reductionist approaches—and Prigogine on dissipative structures, a plethora of studies provided convincing evidence for the existence of physical systems characterized by the spontaneous appearance of properties that cannot be understood, or predicted, from the full knowledge of a system’s constituents.

At a classical scale, an emblematic example of an emergent phenomenon is the turbulence observed in fluids. For instance, in Rayleigh–Bénard convection a fluid is heated from below on a planar horizontal surface, leading to formation of metastable convection cells—known as Bénard cells—which spontaneously break rotational symmetry and self-organize into regular patterns [25]. Turbulence cannot be defined at the scale of a single fluid unit, and it emerges in a wide spectrum of hydrodynamic and non-hydrodynamic systems, ranging from the Earth’s magnetic field to chemical reactions [26]. Remarkably, fully developed turbulence can be reliably described by assuming that the underlying fluctuations cannot be described by a unique scaling exponent but they require a continuous spectrum of exponents, each one belonging to a given fractal set and leading to a multifractal description of the phenomenon [27]. Similarly, chaotic dynamical systems are often characterized by fractal or multifractal organization in space and in time: given their fully deterministic design, their sensitivity to initial conditions is rather unexpected and counterintuitive. In this case, one of the emergent features is the lack of predictability above a certain temporal horizon [28].

Another broad class of spatio-temporal changes in the concentration of chemical or non-chemical substances can be also captured by reaction–diffusion models, widely used to reproduce the pattern formation—known also as Turing patterns [29]—due to the self-organization of travelling waves. Here, an initially homogeneous substance is locally activated by means of reactions while being inhibited at longer ranges: the competition between these two dynamical processes has been widely used to explain morphogenesis in biology [29,30], chemical reactions [31], epidermal wound healing [32], species dynamics [33] and epidemic spreading within a population [34].

Criticality, i.e. the peculiar behaviour exhibited by physical systems at critical points which mark the phase transition between qualitatively distinct regimes, provides another reservoir for emergent phenomena: above a critical point the system can exhibit a feature which disappears once a control parameter, such as the temperature, is tuned below such a threshold. A hallmark of critical phenomena is the presence of long-range correlations within the system’s units: they translate into the lack of a characteristic correlation length, which is typical of power laws. Close to the critical point, we observe a degree of universality: a small number of scaling exponents that can be used to define universality classes able to describe a broad variety of systems which manifest fractal features [35–38]. A widely known emergent phenomenon, such as ferromagnetism, can be understood in terms of the collective behaviour due to the spin-spin interactions of electrons in a material which tend to spontaneously align at the critical temperature, effectively magnetizing the system at large scale. Here, note that ferromagnetism would have no meaning for a system of one particle, since the phenomenon is related to collective behaviour causing simultaneous alignment even in the absence of an external magnetic field. A paradigmatic approach to gain insight about critical phenomena is the Ising model at different dimensions: it has been shown that a simple two-dimensional Ising model with fields is universal, i.e. it can be used to facilitate the physical simulation of Hamiltonians with complex interactions [39] and it has been related to cellular automata [40]. Remarkably, there are systems that do not even need a parameter (e.g. temperature) to be tuned in order to exhibit the scale-invariant organization in space or time, such as in critical systems at phase transition. In fact, such systems dynamically reconfigure their state and spontaneously reach a critical point, which is also an attractor. This peculiar behaviour is widely known as self-organized criticality [41,42] (SOC) and it is characteristic of driven nonlinear systems of many interacting units out of equilibrium [43]: the signature of SOC is the fractal organization in space or time, and it has been observed in biological, ecological, physical and social systems [44,45].

At this point, since most of the systems mentioned so far are non-living, it is worth remarking what is meant by ‘organization’ in open systems out of thermal equilibrium. Here, it is defined by the formation of spatial or temporal (or both) structures that are perceived by an external observer able to measure them in terms of information. On the one hand, this information can be understood as a ‘difference which makes a difference’ [46], which does not allow for an operational definition. On the other hand, the mathematical concept of information as introduced by Claude Shannon [47] allows for different observers to define what it is meaningful to them, leading to a subjective definition of which degrees of freedom are relevant for one’s description of the system and, consequently, the number of possible states used to calculate Shannon entropy, i.e. the average minimum number of binary digits needed to encode a sequence of symbols. It follows that information depends on the observer [48] and, consequently, also the identification of patterns in organizing systems. This is in agreement with William Ross Ashby’s prescription that organization is partly in the eyes of the beholder [49,50]. What can we say, instead, from a thermodynamic perspective? Let us consider a self-organizing system S and an environment E defining, once taken together, a closed universe U=S⋃E. Let $S_{S}$ and $S_{E}$ denote the entropy of the self-organizing system and the environment, respectively. Of course, $\Delta S_{S}/\Delta t>0$ for a purely thermodynamic non-self-organizing system and $\Delta S_{S}/\Delta t=0$ for a mechanical one. Conversely, self-organization requires that the change of entropy per unit of time is negative: i.e. $\Delta S_{S}/\Delta t<0$ for a sufficient amount of time, which requires the entropy of the environment to change as $\Delta S_{E}/\Delta t>0$ not to violate the second law of thermodynamics for the universe U. The presence of irreversible processes contributing to the decrease of system entropy would be balanced by a larger increase in the rest of the universe, i.e. $\Delta S_{U}/\Delta t>0$. Therefore, from a global perspective, one would be forced to disagree with the definition of self-organization, unless one considers the system to perpetually interact with an environment able to supply energy and order, as pointed out by Heinz von Forster in the 1960s [51]. A complementary perspective is that dissipative structures in non-living systems, such as flames or hurricanes, are not true organizational systems since inanimate units cannot organize, but just self-order, themselves [52].

### (c) Emergence in living systems

For living systems, one of the first discussions about self-organization was provided by Erwin Schrödinger in the 1940s, in an attempt to characterize life from a theoretical physics point of view [53]. We can use this perspective as a starting point to briefly review emergent phenomena in living systems, from cells to societies. A heuristic theoretical and computational proof that biological self-organization, or life, is an emergent property of any random dynamical system that possesses a Markov blanket has been given almost a decade ago [54], although a clear mechanism allowing for the transition from an abiotic world to life is still a central problem in research about the origin of life [55–61], especially in prebiotic chemistry [62,63], where primordial reaction networks play a fundamental role, as recently pointed out in the case of spontaneous fine-tuning to environment [64]. At a higher scale, the spontaneous appearance of multiple cell types and their evolution via ecological context, genomic innovation and/or cooperative integration favoured the emergence of multicellular life, boosting biological diversity and complexity: in this context, it has been recently shown that a class of discrete dynamical systems—known as boolean networks and originally introduced to model gene regulatory systems and reproduce their homeostasis and differentiation [65]—can be used to explain cellular differentiation [66]. Note that a better understanding of gene regulatory networks, as well as of the protein–protein and the metabolic interactions, might shed light on the mechanistic rules needed to design, synthesize or reconfigure a minimal organism genome [67], as well as multicellular organisms and living machines [68–72], thus expanding our knowledge of complexity emerging from purely digital systems [73,74].

At a higher scale, the interactions among multicellular organisms lead to unexpected, emergent phenomena that could not be observed or even defined for a single organism. An emblematic example is the ability of *Physarum polycephalum*, the slime mould, to grow adaptive networks able to solve combinatorial optimization problems even if such an organism lacks a nervous system, the one that it is usually assumed to be necessary for such a purpose [75]. For organisms with a nervous system organized as a neural network, additional properties spontaneously appear—e.g. from capacity for generalization, to categorization, error correction, and time sequence retention [76]—although some of these features might not be limited to systems of interacting living units.

Other organisms, as social insects, exhibit a level of organization that leads to swarms, a collective behaviour that can be explained in terms of active matter far from thermodynamic equilibrium: those collectives enable functions that would be otherwise not accessible by each individual in isolation. Herding worms—a physically coupled group of individuals which confers mechanofunctional material properties to the collective [77]—and ant trails [78] and shimmering honeybees clusters [79] are emblematic examples of swarms where a large number of interacting individual units effectively behave as a super-organism where information—from the location of a food source to a migration route—can be transferred without signalling: remarkably, the larger the group the smaller the needed proportion of individuals driving collective decision-making [80,81] (see [82] for a review). Such an amazing behaviour is not limited to insects: from herding [83] to flocking birds [84,85] and schooling fish [86–88], the formation of ordered structures, multistability, mechanical and energetic efficiency are just a few remarkable features of collective states of a large number of individuals which are captured by models grounded on statistical physics [89–93].

### (d) Emergence in social systems

At the scale of humans, interactions among individuals and with the environment are responsible for a variety of emergent phenomena [94]. An emblematic example is provided by social segregation, i.e. the meso-scale organization into clusters, each one characterized by a high level of homophily (e.g. based on gender, ethnicity or socio-economic status). In the 1970s, Thomas Shelling proposed a simple mechanistic model to explain the emergence of segregation: a set of individuals, characterized by a feature with at least two distinct flavours, is homogeneously distributed in space. At successive time steps, each individual is allowed to perform a discriminatory choice based on the tolerance to the abundance of individuals with distinct flavour in his/her neighbourhood: if this abundance is above a predefined threshold, the individual is left free to randomly move to another location. After some time, clusters of same-flavour individuals spontaneously appear, even in the absence of a centralized coordination for their formation [95].

Another interesting phenomenon is population-scale coordination, or social consensus, where collective behaviours spontaneously appear from the microscopic laws of behavioural contagion despite the absence of a centralized organization [96]. The spreading of a behaviour or of an information shares several features with the spreading of an infectious pathogen: epidemic outbreaks are (often temporary) spontaneous phenomena which exploit social interactions to unfold, clustering in space and time [34]. Similarly, a traffic jam cannot be defined at the level of a single unit: be it pedestrians or vehicles, whose dynamic is constrained (or not) to follow lanes and directions, different kinds of congestion usually occur well before the road capacity is reached and such a behaviour can be partially reproduced by microscopic (particle-based), mesoscopic (gas-kinetic), and macroscopic (fluid-dynamic) models [97].

Finally, it is worth mentioning another class of large-scale fascinating complex systems: cities. They are complex from many point of views, consisting of many sub-systems, such as social, economic, environmental ones and combinations of these. It has been shown that a small set of basic principles, operating at a local level, are enough to explain the growth, the large-scale regularities and the scaling laws observed in cities [98–100], once again well captured by models grounded on statistical physics [101,102].

### (e) The role of complex networks in emergent phenomena

A large class of complex systems is characterized by a structure that can be represented in terms of units interconnected by links, which encode one or more kinds of interactions or relationships [103]. Mathematically, such systems can be represented by a matrix or, for more complex systems characterized by multiple types of relationships simultaneously—such as multilayer networks—by a tensor [104,105].

On the one hand, the network backbone itself can represent an emergent feature Σ under some conditions or constraints. This is the case of nested interaction networks that are the result of an optimization principle which maximizes the abundance of species in mutualistic communities [106].

On the other hand, the network backbone can be considered as the starting point for the analysis of structural and dynamical properties Π of a complex system. A pioneering work in this direction unraveled the emergence of power-law scaling in the connectivity distribution of a variety of networks, from biological to technological ones [107]. This discovery led to a plethora of fundamental insights about the behaviour of interconnected systems, from their extreme fragility to targeted attacks to their role in explosive phenomena [108] (see further in this section).

One of the most striking—and ubiquitous—features of empirical complex networks is the emergence of a mesoscale organization, such as hierarchical [109,110] and/or modular [111–113] structure, which has been linked to efficiency in information exchange, functional segregation and integration [114–123]. Networks exhibit other emergent features, such as latent geometry [124] or distinct flavours of multilayer organization, from interdependence [125] to multiplexity [104,105,126–129].

Uncovering structural features of networks is a necessary step towards understanding the function(s) of the underlying complex system they are representing, since functionality interlaces with the dynamics of or on the network. For instance, the network counterpart of the Anderson localization has been reported [130]. In the case of networks of oscillators, the collective phenomenon of synchronization spontaneously emerges if the coupling between units is above a critical threshold [131–134], with a variety of phenomena ranging from explosive behaviour in scale-free networks [135] to new types of collective states emerging from coupling synchronization dynamics with swarming behaviour, like in swarmalators [136], or network dynamics [137]. Similarly, some critical properties—such as the emergence of metacritical points—start to depend on the way distinct dynamics are coupled together, such as in interacting spreading phenomena on the top of simple or multilayer networks [129,138–140].

As mentioned above, the network representation of empirical systems usually supports dynamical processes on them [141]. For instance, in the power grid, the electricity is generated and delivered, transportation networks sustain a flow of people and goods from one place to another, users navigate the content in the World Wide Web through hyperlinks, information or a pathogen spreads in online and offline social networks via friendship and acquaintance ties, etc. To some extent, the sustained network-wide functionality can be seen as a robust emergent phenomenon that dodges disrupting events—such as errors, random failures or attacks—in the individual units of the network. Thus, functionality, robustness and resilience can be seen as complementary emerging properties of a system.

The link between network functionality and robustness has been actively investigated due to its societal impact, for instance, at the infrastructural or ecological levels. Bare-bones approaches have looked at the size of the largest connected component of the network, assuming it is the most functional part, when the original system is perturbed with the removal of a given fraction of nodes or links. This is the reversed process of adding nodes or links in an initially empty network and track when a macroscopically functional structure emerges. Both processes are completely equivalent in the absence of hysteresis loops. Percolation theory [142] turns out to be useful in this case, as it provides a set of concepts and analytical and computational techniques suitable to describe this functional-to-nonfunctional transition. Diverse intervention protocols have been proposed to dismantle the system: random uniform selection of nodes to model unexpected disruptions [143], while informed interventions can be seen as targeted attacks. Examples of the latter included making use of both topological [143–145] and non-topological information [146]. Moreover, the emergence of the functional structure can be achieved in an explosive, abrupt manner via the design of topologically sophisticated rules [147–149] or via the inclusion of interdependencies [150,151].

Another realistic approach is to consider how networks respond to cascading failures. These processes are characterized by an initial stressor—located in a small region of the network—that is able to spread and impact large portions of the network. Different propagation rules have been proposed, whose characteristics depend on the system one is trying to model (see [152–157]). Of particular interest are the cascades spreading in multilayer and interdependent structures, as it has been shown that they could suddenly collapse, thus making it difficult to identify early signals of fragmentation [125,158]. Instead of a malfunction that spreads, one can also consider how the exploration or navigability properties of walkers are affected when some of the network components are corrupted. It has been reported that these characteristics are greatly impacted by the walk strategy and topological properties [159].

## 4. Defining emergence from a mathematical perspective

David Chalmers clearly distinguishes between two types of emergence: *weak* and *strong*. A phenomenon at a high scale is *weakly emergent* with respect to a lower scale if, given the laws governing the latter, the patterns observed in the former are unexpected, but they can be deducible in principle from advanced calculations and/or computation. If such a deduction is not possible even in principle, then the phenomenon is *strongly emergent* [160]. A similar distinction was already present in the work of Mark Bedau, who identified two hallmarks of emergence where phenomena are either (i) somehow constituted by, and generated from, underlying processes; or (ii) somehow autonomous from underlying processes [161]. The existence of strongly emergent phenomena would require new fundamental laws of nature for their explanation. In fact, it is argued that only the weak emergence is scientifically relevant, consistent with materialism and metaphysically innocent to provide a ground for a science of complexity [161]. The possibility that a simple initial configuration might evolve into unexpected patterns allows us to overcome the reductionist approaches while preserving the possibility for rich phenomena at distinct levels of explanation and, consequently, an ultimately physicalist picture of the world [160]. We capitalize on these arguments to rationalize an operational definition.

**Figure 1.**
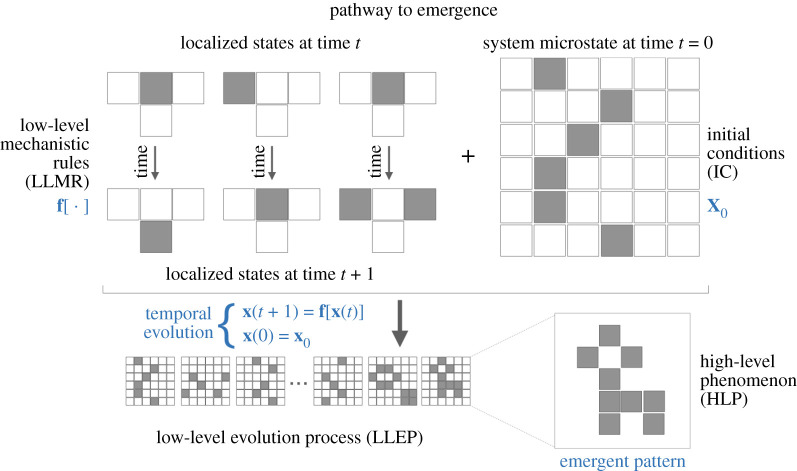
A complex system consists of interconnected units for which a set of local mechanistic rules for hanging over time is assigned (top left). An initial condition for such a system is given (top right) and the system evolves according to its microscopic rules until an emergent pattern is observed. The reader might identify an analogy with the well-known Game of Life, a cellular automaton proposed by John Conway in the 1970s [162] for which, more recently, quantum versions are being explored [163]. Note, however, that we do not require the state of the units to be binary, or even discrete. The analogy can be used to better understand the rich basin of phenomena that can take place when a few microscopic rules and adequate initial conditions are considered: in fact, it should be noted that not all possible LLMR and IC lead to HLP. See the text for further details. (Online version in colour.)

Let a system S be made up of a finite number of many units u that interact among them and/or with the environment. Emergence is the apparition of system-wide properties or qualities that are not present individually in the units but have their origin precisely in the interactions. Let us assign a set of simple low-level mechanistic rules (LLMR) f[⋅] that allow our units to change locally. If we indicate the state of the system at a specific time t by x(t), then such rules can be encoded into an evolution equation that can be discrete or continuous in time. For simplicity, let us consider a discrete-time evolution, as schematically shown in figure 1, and an initial system state which is mathematically represented by

4.1 $$\begin{array}{rl} & \text{x}(t+1)=\text{f}[\text{x}(t)]\\ \;\mathrm{and}\; & \text{x}(t=0)={\text{x}}_{0}, \end{array}\}$$

where ${\text{x}}_{0}$ defines initial conditions (IC). The microscopic evolution of the system is defined as a low-level evolution process (LLEP) and, after some time, it will lead to a stable or metastable emergent pattern, i.e. a high-level phenomenon (HLP). Note that we did not specify if f[⋅] is deterministic or stochastic, and if the system is open or closed, since any combination of them is plausible, in principle. In fact, if the system is open and the dynamical rules are deterministic, the contingencies of the flux of parts and states through S provide additional external conditions, whereas if the system is closed there is only one external condition, i.e. the initial one. If the dynamics is stochastic, then accidental effects provide additional external conditions. It is clear as a full knowledge of S, f[⋅], ${\text{x}}_{0}$ and eventual external conditions might allow, through computational analysis, to evolve microstates and observe unexpected macrostates. Summarizing:

— **Non-emergent phenomena:** knowledge of LLMR and IC allows us to deduce expected HLP;
— **Weakly emergent phenomena:** knowledge of LLMR and IC allows us to deduce unexpected HLP through computation (e.g. simulations);
— **Strongly emergent phenomena:** knowledge of LLMR and IC does not allow us to deduce HLP even in principle.

Note that the above classification applies well for two distinct types of emergent phenomena we have been discussing: (1) the ones where the system Σ itself emerges from its constituents, as for the spontaneous organization into structural backbones mentioned in the previous section; (2) the ones where macroscopic properties Π emerge because of the existence of a system (e.g. superconductivity or the robustness of a network to random disruptions). Such a hierarchy between emergent phenomena, involving large-scale structures and properties, can be used to better understand the results of the studies published in this theme issue.

## 5. Summary of the theme issue

At this point, the general reader should be familiar with the concept of emergence. In this section, we briefly introduce the papers in this collection: each manuscript has been developed independently from the others but at the same time it is connected to them to allow for the exploration of emergence from a multidisciplinary and interdisciplinary perspective. To some extent, the emergent result is this theme issue. They are organized, here and in the issue, as follows: we first present the theoretical contributions dealing with the problem of emergence itself, then we move to the contributions about the emergence of specific phenomena in different contexts. The latter are introduced according to their scale, from smallest (quantum realm) to largest ones (epidemics).

The theme issue starts with the article *Emergence and algorithmic information dynamics of systems and observers* by Abrahão & Zenil [164]. The authors deal with the problem of asserting whether or not a phenomenon can be considered emergent, from the viewpoint of computation theory. Identifying the act of observing as a mutual perturbation between the system and the observer, they find that the emergence of algorithmic information is dependent on the observer’s formal knowledge but robust to other subjective factors. Additionally, they prove that emergence becomes observer-independent if it increases in an unbounded and rapid fashion, and two examples are studied to illustrate this phenomenon.

It is followed by the article by Rosas and collaborators, *Greater than the parts: a review of the information decomposition approach to causal emergence* [165], which offers an accessible and rigorous review of a recently developed formal theory of causal emergence. This theory is based on information decomposition, where emergence is considered a property of part-whole relationships within the system under study. They present a mathematical background, the key principles of the theory and several case studies, both from empirical data and synthetic simulations, that demonstrate the applicability of their approach.

In the search for a solid and convincing theory of emergence, Varley and Hoel’s contribution, *Emergence as the conversion of information: a unifying theory* [166], overcomes the traditional dichotomy between strong and weak emergence and, aiming at finding a formal theory of emergence able to identify the intrinsic scale of function of complex systems, propose a mathematical framework in which emergence is identified with information conversion across scales. They base it on information theory and successfully apply it on a model system of Boolean networks.

In *Emergence of functional information from multivariate correlations* [167], Adami and Nitash draw a mapping between the multivariate correlations within a symbolic sequence, such as the nucleic- or aminoacid ones, and the functional information encoded on it. Their model-free approach is tested in the largest known computational genotype–phenotype map, in which they are successful in distinguishing functional from non-functional sequences.

We have already discussed that one necessary condition to find emergence is to have a system made of many small interacting sub-systems. In *Strengthened second law for multi-dimensional systems coupled to multiple thermodynamic reservoirs* [168], David Wolpert studies the stochastic thermodynamic properties of such systems, under the only requirement that they evolve according to a continuous-time Markov chain. Lower bounds for the entropy production are derived, hence offering a strengthened version of the second law of thermodynamics.

The critical phenomena observed in phase transitions are a paradigmatic example of how local interactions lead to system-wide effects. In their article, *Emergent entanglement structures and self-similarity in quantum spin chains* [169], Sokolov *et al*. provide a thorough characterization of this in the quantum realm. By means of tools borrowed from complex network theory, they unveil new emergent phenomena in spins interacting through the XX model of magnetism, such as an entangled community structure, topological instabilities and self-similarity in the entanglement network.

Moving up to higher scales, we pass from spins to molecules and metabolism. In Nader, Sebastianelli and Mansy’s Opinion Piece, *Protometabolism as out-of-equilibrium chemistry* [170], the authors argue for the need to explore the role played by prebiotic energy sources as a possible explanation of the origin of metabolism. They put the focus on the out-of-equilibrium chemical properties, advocating for non-hydrothermal vents as regions of the early Earth that could be able to provide the necessary energy to sustain the first protocells.

Still at the molecular scale, Xavier and Kauffman’s article *Small-molecule autocatalytic networks are universal metabolic fossils* [171] focuses on the emergence of early metabolism. They investigate small-molecule reflexively autocatalytic food-generated networks, proving that these structures can be generated from all the hitherto annotated prokaryotic metabolic networks in the KEGG database. The results based on the analysis of these networks yield the striking conclusion that molecular reproduction started much earlier than the last universal common ancestor.

Molecular function emerges from molecular evolution. In the review article, *The simple emergence of complex molecular function* [172], Manrubia guides us through some of the mechanisms that facilitate this evolution, such as phenotypic bias, genotype-to-phenotype redundancy, among others. When all these mechanisms are taken together, molecular complexity seems the most natural outcome.

Moving to larger scales, we arrive at neurons and the brain. It is well-known that brain states, both healthy and altered, can be characterized by complex emergent spatio-temporal patterns. In *Understanding brain states across spacetime informed by whole-brain modelling* [173] Vohryzek and coauthors embrace the idea of considering the human brain as a complex system, and they offer a review on how these patterns can be mapped and modelled via non-invasive imaging and whole-brain modelling, with a focus on depression and psychedelics.

In the brain, we also find synchronization phenomena. Buendía and coauthors provide in *The broad edge of synchronisation: Griffiths effects and collective phenomena in brain networks* [174] a thorough characterization of the rich dynamical repertoire that arises in brain synchronization when it is combined with a minimal dynamical model of neural activity with empirically observed properties of brain connectivity, such as hierarchical-modular and core-periphery structures. They reveal the emergence of complex collective states with flexible levels of synchronization, which is a necessary step towards a better understanding of the functional capabilities of brains.

Saeedian *et al*.’s article *Effect of delay on the emergent stability patterns in Lotka–Volterra ecological dynamics* [175] finds itself at the ecosystem scale. They tackle the problem of ecosystem stability when the realistic feature of delayed interactions between species is incorporated in a generalized Lotka–Volterra model. They provide analytical and numerical results as a function of the delay strength, and report a detrimental effect on the ecosystem stability as delay increases. At a critical value of the delay, oscillatory states emerge, which is a dynamical regime that could not be predicted by standard linear stability analysis.

Peters and Adamou tackle the problem of cooperation, understood as resource sharing between system units, such as cells, animals, humans, institutions, etc. In the opinion piece, *The ergodicity solution of the cooperation puzzle* [176], they propose a simple model where this behaviour, which might seem not appealing to one of the cooperators, can arise even if the classical assumptions of reciprocity or the existence of net benefit between cooperators are not met, as far as the resources follow a noisy multiplicative growth. Thus their model becomes a candidate to explain cooperation in many real settings and provides a baseline for behavioural comparison.

Our journey across scale reaches the human one at this point. In the review, *A research agenda for the study of social norm change* [177], Andrighetto and Vriens provide a complete overview on social norm change. Indeed, interactions among social agents are the basis on which norms emerge and develop, hence being an interesting tool to tackle collective action problems. They critically discuss how to identify social norms, how to establish causal effects, how norm change is linked to tipping point dynamics, and outline future research problems.

Humans do not only interact directly among us, but also with technological devices. Brinkmann and coauthors shed light on the role that the interaction between humans and algorithms might play in shaping the emergent properties of cultural evolution. In *Hybrid social learning in human-algorithm cultural transmission* [178] they propose a set of six hypotheses, related to the improvement of collective performance tasks via (hybrid) social learning, that are tested in an experimental set-up. Their empirical findings highlight the importance of biases: even if an algorithm aims to aid humans, the provided information can be quickly lost in successive human–human interactions due to, precisely, human biases. Closing the theme issue, we find the article *Emergence of protective behaviour under different risk perceptions to disease spreading* [179], by Khanjanianpak *et al*. The authors address the problem of how different behavioural responses emerge when a population is exposed to a given risk, when the latter is heterogeneously perceived within the population. They focus on the timely problem of the adoption of protective measures, such as social distancing, social distancing, etc. during the course of a disease spreading.

## 6. Conclusion and outlooks

Since the first discovery of emergent phenomena a plethora of phenomenological evidence has been provided to show that they are ubiquitous, from quantum to classical physical systems, from non-living to living ones. Nevertheless, we envision outstanding challenges and a rather exciting agenda for both fundamental and applied research on emergence in the near future.

On the one hand, many emergent processes exhibit similar properties—e.g. cluster formation in critical physical systems consisting of many particles, and in social processes—and domain-specific peculiarities. Unraveling the building blocks of an emergent phenomenon in space, time, or both in space and time, is still an open problem: while similarities across disciplines suggest the existence of a few generative rules, thus increasing the likelihood of finding such mechanisms, the advances in this direction might be slowed down by domain-specific microscopic rules which might be difficult to reconcile within a comprehensive vision. It is worth remarking here that it is still not granted that such a comprehensive picture exists or if it useful at all. Moreover, even the inverse problem is an open challenge: predicting the macroscopic outcome(s) of a process, given the microscopic rules governing a system’s units might lead to the discovery of a new kind of universality class. Recent advances in the latter are related to programmable pattern formation, for which there are interesting applications to the case of cellular systems with local signalling [180].

On the other hand, the outcome of such an understanding at a fundamental level might open the door to countless applications in physics, biology and engineering, to mention a few. One might be able to design physical systems exhibiting (weakly) emergent properties, such as robot swarms able to self-assemble [181], self-repair and exhibit high robustness to internal failures [182], with the ultimate goal of engineering complex systems able to perform specific tasks and achieve human-designed goals. The basin of applications ranges from medicine to cybersecurity.

In a nutshell, in such robot swarms each unit communicates or interacts only locally with its neighbours: all together they are able to generate flexible and scalable collective behaviours without relying on external infrastructures or centralized control, actively adapting in response to stimuli from the environment in which they are embedded. The interested reader is referred to [183].

An emblematic example is given by large flocks of autonomous drones that seamlessly navigate in confined spaces [184]. Furthermore, it has been shown that robophysical systems known as ‘smarticles’—planar ensembles of periodically deforming smart, active particles—are able to generate endogenous phototaxis, a kind of locomotory movement occurring when a collective of organisms moves in response to the presence or absence of light, thus providing a model to develop internal mechanical interactions to perform tasks without a centralized coordination [185]. As biological systems combine microscopic stochastic components to achieve a desired macroscopic function, such as cell migration in morphogenesis, tissue repair, and cancer [186], robot swarms might achieve a similar behaviour. Recently, it has been shown that robot swarms are able to mimic the behaviour of labour division in ant colonies [187] and herding worms [77], undergoing shape transformations which make the system more robust to thermal stress or more energetically efficient.

In computer science, swarm learning, a machine learning explicitly based on decentralized approaches that rely on networks of learners, has outperformed the standard federating learning. Remarkably, it has been shown that it achieves better results than scenarios in which each node in the network learns separately [188].

Such exciting advances in technological applications of self-organizing artificial systems, at both software and hardware level, might be employed to detect threats in IT systems and build robust security layers, as well as to accelerate the recovery of networked systems and infrastructures—e.g. telecommunications, power, water management, supply chain, so forth and so on—or reduce the gap to precision medicine with personalized clinical treatments.

Wrapping up, there is great promise in unraveling the principles of emergent phenomena which could potentially find groundbreaking application in material science, nanotechnology, medicine, engineering and computer science. If we are forced to summarize the concept of emergence by means of figurative language, we can safely assess that there were no lasagne encoded in the Big Bang.

## Data Availability

This article has no additional data.
